# Reconciling *art* and *science* in the era of personalised medicine: the legacy of Georges Canguilhem

**DOI:** 10.1186/s13010-023-00133-9

**Published:** 2023-05-24

**Authors:** Gianmarco Contino

**Affiliations:** 1 Von Hügel Institute, University of Cambridge, Cambridge, UK; 2 Institute of Cancer and Genomic Sciences, University of Birmingham, College of Medicine, Vincent Drive, Edgbaston, Birmingham, B152TT UK

**Keywords:** Epistemology (K01.468), Medical Philosophy (K01.752.667), Experimental Medicine (H01.770.644.145)

## Abstract

**Background:**

Biomedicine, i.e. the application of basic sciences to medicine, has become the cornerstone for the study of etiopathogenesis and treatment of diseases. Biomedicine has enormously contributed to the progress of medicine and healthcare and has become the preferred approach to medical problems in the West. The developments in statistical inference and machine learning techniques have provided the foundation for personalised medicine where clinical management can be fully informed by biomedicine. The deployment of precision medicine may impact the autonomy and self-normativity of the patients. Understanding the relationship between biomedicine and medical practice can help navigate the benefits and challenges offered by precision medicine.

**Methods:**

Conventional content analysis was applied to “Le Normal and le Pathologique” (Canguilhem G. The Normal and the Pathological. Princeton: Princeton University Press; 1991) and further investigated with respect to its relationship with *techne* and precision medicine using PubMed and Google Scholar and the Standford Encyclopedia of Philosophy to search for the following keywords singularly or in combination: “Canguilhem”, “*techne*”, “*episteme*”, “precision medicine”, “machine learning AND medicine”.

**Results:**

The Hippocratic concept of *techne* accounts for many characteristics of medical knowledge and practice. The advances of biomedicine, experimental medicine and, more recently, machine learning offer, in contrast, the model of a medicine based purely on *episteme*. I argue that Canguilhem medical epistemology establishes a framework where *episteme* and data-driven medicine is compatible with the promotion of patient’s autonomy and self-normativity.

**Conclusions:**

Canguilhem’s medical epistemology orders the relationship of applied medicine with experimental sciences, ethics and social sciences. It provides guidance to define the scope of medicine and the boundaries of medicalization of healthy life. Finally, it sets an agenda for a safe implementation of machine learning in medicine.

## Background

Starting from the eighteenth century, experimental medicine has use instead elevated medicine to the rank of experimental science. Biomedicine, i.e. the application of basic sciences to medicine, has become the cornerstone for the study of etiopathogenesis and treatment of diseases. Biomedicine has enormously contributed to the progress of medicine and healthcare and has become the preferred approach to medical problems in the West. Biomedicine has provided greater control and power of prediction over medical events. As a result, the scope of medicine widened beyond disease treatment to include disease prevention and optimization of health and enhancement of human physiology; a process known as medicalization or, more appropriately, biomedicalization of healthy life [8].

With few notable exceptions (e.g. epidemiology, social medicine etc.), the endpoint of medical practice is concerned with the decision making to care for a specific patient (medicine-in-particular). Biomedicine derives experimental evidence from averages (groups of patients, animal models, cell lines) and informs clinical decision making. Developments in statistical inference and machine learning techniques provided the foundation for personalised medicine that promotes a model of medicine where biomedicine and clinical management coincide. This superimposition is highly problematic: biomedicine remains a “medicine-in-general” even when it claims to seek “precision” or “personalized” medicine [12]. There is a qualitative gap between medicine-in-general and medicine-in-particular: the latter cannot be achieved through incremental adjustment of the first one. Understanding the nature of this qualitative gap can help navigate the benefits and challenges of thriving scientific progress, order the hierarchical relationship of medicine with experimental sciences, ethics and social sciences, define the field of application of medicine and preserve society from potentially harmful forms of medicalization. To do so, the paper explores the Hippocratic and Aristotelian idea of *techne* and delineates the epistemic principles that differentiate medicine from medical science. Finally, I attempt a reconciliation leveraging on Canguilhem’s analysis of medical epistemology that incorporates the positive epistemology of medical science into a more articulate view of the normal and pathological.

## Methods

Conventional content analysis was applied to “Le Normal and le Pathologique” [4] and further investigated with respect to its relationship with *techne* and precision medicine using PubMed and Google Scholar and the Stanford Encyclopedia of Philosophy to search for the following keywords singularly or in combination: “Canguilhem”, “*techne*”, “*episteme*”, “precision medicine”, “machine learning AND medicine”.

## Results

### Quantification and determinism in medical sciences

Human pathophysiology can be investigated at the phenotypic, cellular and molecular level through experimental methods. Using disease models and biospecimens, medical science can test hypotheses, collect and analyse controlled experimental data. The laboratory is the ideal setting to investigate the biology of health and disease, identify novel treatments and advance medical knowledge. The most fundamental feature of laboratory medicine, as thought by its founder Claude Bernard, is quantification: each biological phenomenon can be reproduced in laboratory models, measured and computed. Claude Bernard was an assertive determinist. He believed that “*the real and effective cause of a disease must be constant and determined*, that is unique; anything else would be a denial of science in medicine” [2].

Clinical medicine has also developed quantitative tools to assess clinical treatments under the pressure of standardising treatments and outcomes. Evidence Based Medicine pursues a medicine based on the strongest evidence available and promotes randomized controlled trials as a tool to reduce bias and improve the quality of medical science. Bernard’s deterministic view proved extremely influential not only in experimental medicine but also in Evidence Based Medicine and most recently in Precision Medicine. Not surprisingly these fields share Bernard’s controversial relationship with statistics. Bernard rejected the use of statistics acknowledging that “a very frequent application to biology (is) the use of averages (…) which may give only apparent accuracy” [2]. He soon realized that experimental medicine cannot model the individual patient: “no two patients are ever exactly alike,their age, sex, temperament and any number of other circumstances involve differences, with the result that the average, or the relation deduced from our comparison of facts, may always be contested. But I cannot accept even the hypothesis that facts can ever be absolutely alike and comparable in statistics; they must necessarily differ at some point, for statistics would otherwise lead to absolute scientific results, while they can actually show only probability, never certainty. I acknowledge my inability to understand why results taken from statistics are called laws; for in my opinion scientific law can be based only on certainty, on absolute determinism, not on probability” [2].

Nonetheless statistics has become the gatekeeper of scientific validation when it comes to complex biological systems. To pin down a molecular mechanism we need multiple observations and each of these observations takes in consideration a number of molecules, cells, events, etc. that must be interpreted in statistical terms. Statistical modelling has taken advantage of the exponential availability of computational power and novel methodologies: its applications are at the core of data science, modeling of biological events, machine learning and prediction of health and outcomes. The power of these techniques has generated a new wave of determinism. We have come to a statistical positivism where probabilistic outcomes are looked at with the same deterministic outlook of Bernard.

### Medicine-in-general and medicine-in-particular

The inference from medicine-in-general to medicine-in-particular, or, in other words, from averages to individuals remains a major epistemic problem. Medicine-in-general has several characteristics of a positive science: quantification of empirical observations, collection of data, statistical analysis, description of laws, prediction of responses. To do so, however, disease must be objectively measured and quantified against a statistical normality that is subjected to variation across populations and time. Because of this variation, one could argue that disease is not an objective condition but rather a conventional one. We often see novel conditions or diseases being described depending on the availability of diagnostic criteria (e.g. chronic fatigue syndrome), and others reverted to normality based on changing social paradigms (e.g. homosexuality). Strikingly, neither medically defined disease nor normality define a universally accepted concept of health: a subject can experience lack of health even in absence of objectively measurable disease and another can feel completely healthy even in presence of a measurable disease or disability. Health and pathology can coexist, while the experience of disease only exist with a reference to the specific patient in a specific time and environment, but not in absolute terms. Consequently, the transition from medicine-in-general to the single patient requires adjustment that cannot be resolved within a positive epistemology.

### The different epistemology of medicine and biomedicine

To understand the roots of medical epistemology it is useful to go back to the original description in the Corpus Hippocraticus. Here the author of “The Art” (*Peri Techne*) describes medicine as an art (*techne*) in partial opposition to science (*episteme*) [9, 18]. This manuscript is possibly the first attempt at general epistemology bequeathed to us by antiquity [22]. *Techne* has often been pictured as a mean to deal with the lack of satisfactory *episteme*, in the conviction that it would only be a matter of time before science fills the knowledge gap. *Techne* is instead a more profound idea that lies at the root of western medicine and defines its limits and scope [19]. *Techne* is characterised by being confined to a specific subject, targeted to a precise end and carrying a useful result. In “The Art” the author translates those characteristics to the art of medicine according to these principles: 1) the subject of medicine should be confined to the diseased human body; 2) the end of medicine is to heal and to help the patient; 3) medicine should provide the relief of suffering caused by disease but refuse to treat incurable disorders; 4) the product of medicine is not health-in-general but concerns a specific result (*ergon,* “production” which results from techne) for the patient [18, 19]. *Techne* and *episteme* are not mutually exclusive: *Techne* requires the knowledge of general principles but also implies the aim of making, doing or, in the case of medicine, to help. Thus, investigation and understanding are not enough to define medicine. In the Nicomachean Ethics, Aristoteles points out that “Science is the knowledge of that which exists of necessity, is eternal (knowledge of that which cannot be any way other than the way it is because it is unchanging), and can be learned (1139b20-26)”, while Art “ is concerned with the process of coming into being (1140a10). Art (…) operates in the sphere of the variable”. [N.E. 1140a1-23]. Therefore, *Techne* is not only subject-specific but also time-specific. Medicine-*techne* is there to help a specific patient at a specific time of their life. Patient’s condition is ever-changing and so is the temporary and incomplete nature of medical knowledge.

In the *Nicomachean Ethics*, Aristotle argues that the physician does not study health as such but human health – even the health of “this human” because it is individuals that he cures (1097a10-15) [14]. Medicine is therefore *episteme* of health but primarily *techne* (craftsmanship) of individual health. Medicine only exists by virtue of its goal (*ergon*) which is the helping an individual person who is suffering. Again, we find in Aristotle the theme of contingency as a main attribute of the medical art (*techne iatrike*). While *episteme* is concerned with the necessary prime principles, *techne* deals with the ever-changing nature of human conditions, possibilities and opportunities. Aristotle seems to warn us against the possibility of a complete understanding of health when it comes to an individual patient; a limitation that remains despite the sophistications of the *episteme*. In fact, not even science is a safe harbour to our aspiration to completeness. We accept that medical knowledge is incomplete and provisional and requires the involvement of the patient, to understand his perspective and his autonomy for its actuation.

Without any ambition to provide a historical account of the theme, more contemporary authors have further elaborated on the idea of techne taking in account the ever-increasing impact of technology in human life and knowledge. In his essay on “The question Concerning Technology” [16], Heidegger connects techne to the revelation of something in the realm of reality. Again, the idea of contingency is strictly connected to the manufacture (*poiesis*) but entails the responsibility of making it happen or causing [16, 22, 31]. Heidegger goes back to the Greek etimology for cause, aitia, which means “to make present”, “to occasion” in the sense of bringing something that was not present before into time and space. Thus, *techne* is the way to reveal the truth (*aletheia* in greek means to truth but also revelation) that is bound to the human experience of life [10, 32]. There is a two-way relationship between *techne* and truth. *Techne* gives shape to our ideas, but we can better shape our ideas if we build better technologies which are the tools of techne. In other words, techne is not only the instruments to deal with the ever-changing nature of human conditions but also the cause of the changes, hence the responsibility. *Techne* is at the core of our existence as human being: as we achieve our goals, we also shape reality and steer the future of humanity [3, 16, 22]. One could argue that techne provides the foundation not just for medical epistemology but also medical ethics. Along this line, Dewey’s pragmatism captures a critical aspect of medical epistemology when it states: “what measures [knowledge’s] value, its correctness and truth, is the degree of its availability for conducting to a successful issue the activities of living beings [11]”.

Finally, we should ask whether a pure episteme is even possible in medicine. Using Dewey’s pragmatism, we could argue that reasoning is always permeated with both feelings and practical exigencies. and that knowledge is bound to sensations and sensations are not a separable content of consciousness. Our brain, senses and body are nonetheless the technology of episteme. In other words, episteme needs tool that are subjected to the epistemology of techne even when they try to achieve pure episteme. A view that has been corroborated by the development of neuroscience and influenced the work of philosophers like Polanyi with the importance given to tacit knowledge which plays a role in every medical encounter [17].

### Precision medicine is a form of medicine-in-general

Precision medicine is a medical model whose goal is to tailor prevention, diagnosis and treatment to the individual patient based on genetic, clinical, environmental and research data [24]. Precision medicine is data intensive and builds on the advances of machine learning applied to large training sets of patient and population data. It has been shown that machine learning (in particular deep machine learning based on neural network algorithms) can overperform the diagnostic accuracy and outcome prediction of expert clinicians [1, 13, 23, 32]. In practical terms clinicians and patients will be able to make decision based on more accurate knowledge of the benefits and risks they face. In other terms precision medicine allows to classify individuals into subpopulations that differ in their susceptibility to a disease or their response to a specific treatment [25]. This is likely to be the most realistic outcome of precision medicine and, in this sense, it could be merely seen as an upgrade over evidence-based medicine approaches based on large population studies.

Nonetheless, the power of deep machine learning and the exponential capacity to acquire health data including sequencing, imaging and exposures may have deeper implications for medicine. There are technological and policy challenges linked to the use of deep machine learning in medicine. Many of those are actively being addressed although the deployment of machine learning is moving faster than its fixes. A less debated risk comes from the positivist outlook around precision medicine and machine learning. The view that precision medicine will eventually replace the need for Aristotelian *techne* in medicine is set to fail, not dissimilarly from what already experienced with evidence-based medicine. On the other hand, the scale at which machine learning-driven precision medicine is going to change our relationship with health and disease cannot be discounted. A key aspect is the intrinsic black box nature of large neural network that makes difficult to explain how certain preditctions are reached. In addition, several algorithms will function at different steps in the information workflow (e.g.: signal processing of diagnostic machines, clinical data extraction and integration, disease specific outcome prediction), each introducing black boxes and possible biases. Importantly, there will be value considerations that are embedded in the research data, study design and algorithms that are not explicit. This latter problem has been convincingly shown already in evidence-based medicine and it is only made more vicious by the difficulty to trace back information. Eventually, we may need to accept highly accurate prediction that are unexplainable and oracular albeit probabilistic. When we misinterpret probabilistic and incomplete models with a deterministic approach we constrain patient’s autonomy and surprisingly interfere with the outcomes.

The myth of Oedipus that Sophocles has rendered in the Oedipus Rex, is a powerful tale of this conundrum. Laius, king of Thebes learns from the oracle of Delphi that the son he will have from his wife Jocasta will murder him and marry Jocasta. To avoid this from happening Laius rejects Jocasta, who in turn tries to kill the son she secretly had from Laius: Oedipus. Oedipus is saved by a shepherd and brought to the court of Polybius, king of Corinth, where he is brought up as the alleged son of the king. When Oedipus learns from the oracle that he will kill his father and marry his mother, he escapes to Thebes. But avoiding this prophecy only leads to its fulfilment; on the way to Thebes he engages in a fight with Laius, his real father, and once in Thebes he marries Jocasta. The wisdom of the Oedipus warrants that predictions and information alter our status, sometimes making the outcome more likely in unpredictable ways. A strong rationale for predictive genetic and non-genetic testing of multifactorial diseases such as cardiovascular disease, type 2 diabetes, obesity, cancer or depression is that awareness will trigger behavioural changes. However, meta-analyses showed that communicating DNA-based risk estimates does not motivate risk reducing behaviour [20], instead experimental evidence suggests that knowing the risk independently alter physiology, subjective experience and behaviour in ways that may exacerbate actual risk [29].

It appears that, at a time where medicine is drive by fast paced technoscientific developments, we need even more *techne* to navigate the complex nature of information and the consequences on individual health and disease. Eventually, precision medicine will only make more evident that *techne* is not mutually exclusive but rather the epistemological toolset to apply *episteme* to medicine. *Techne* is therefore a powerful tool that allows the qualitative transition from medicine-in-general to medicine-in-particular and help navigate the limitations of medicine and its relationship with science.

### Medicine as a personal history: reconciling medicine-in-general with medicine-in-particular

The transition from medicine-in-general to medicine-in-particular is a qualitative step that takes into account the irreducible diversity of every person. The epistemology of medicine-in-particular has been explored by George Canguilhem in his treatise “On the normal and pathological”. Canguilhem argues that disease is not a quantitative degree in the spectrum of health, instead health and disease are evaluative terms that signal qualitative distinctness [4, 28]^1^^,^^2^^,^^3^ Canguilhem’s views are radically holistic. He shares with Bernard the preoccupation of using statistics to understand individual phenomena, but he resolves the question by denying the possibility of reducing health and diseases beyond the concrete human beings as independent total wholes. He rejects that organs or cells can be diseased, except in the most metaphorical senses [28]. This notion may appear obsolete or counterintuitive in an era where molecular defects causing diseases are constantly being discovered and cell and organ transplantation can cure diseases. The conclusion is that "if one wants to define disease, it must be dehumanized" [73, 6.22–3]; and more brutally, "in disease, when all is said and done, the least important thing is man" [73, 6.22–4] [4]. As noted by Spicker, any reference to particular diseases in particular parts of the patient’s body is “derivative talk”; the diseased liver is only “diseased’ because it is intimately bound to the suffering patient living within his or her full environmental and socio-cultural context [4–6, 29].^4^

Canguilhem sees health as the ability of the organism to adapt to challenges posed by the environment, to create new norms for new settings [21]. For him the normality is measured by the adaptability of the individual [21]. In other terms the individual is normative to himself. Consistently, the role of medicine is not to establish norms but rather help individuals to navigate their own norms relative to the changing environments and experiences of life. Far from the political implications developed by his scholar Foucault, Canguilhem’s view interrogates on the relationship between medicine and individuals and between medicine-in-general and medicine-in-particular.

Canguilhem’s medicine is not compatible with a medicine-episteme which is, by definition, normative at least in a statistical manner. Medicine-episteme establishes norms and defines laws that allow prediction and therefore control over events including individual lives (not the particular individual, but an averaged individual or an algorithmic individual). Canguilhem medicine exposes the Achilles heel of medicine-episteme. There is no continuity between medicine-in-general (even in its personalized forms) and medicine-in-particular. In fact, the practice of medicine is necessarily and primarily a medicine-in-particular and this is a universal statement. Every medical act is personal, time and space bound, and unique, in line with medicine as techne. The particular story and the autonomous choice of the patient collapse the probabilistic space of medicine-in-general to a singularity [4–6].

The first consequence of such a view is to re-establish a hierarchy of knowledge: medicine is performative while medical science is informative. The first one accommodates incompleteness the latter fights it. Medicine recognises the interplay of multiple epistemologies and knowledge (e.g.: science, ethics, theology, social sciences), instead medical science has little play around a dominant epistemology. Medicine and medical science are incommensurable in the sense that medicine is a meta-system that can incorporate elements of medical science. Canguilhem recognises that medicine and life sciences cannot be forced in the epistemology of physics which has been used as the gold standard for all sciences. This operation allows him to recompose the threads of the different contributions that have enriched the episteme-techne debate and medical epistemology since Aristotle. Canguilhem blends the influence of Bernard, Leriche, Heidegger, Dewey, Polanyi and Husserl among many others remaining an Aristotelian at the bottom [6, 15]. In Foucault words, Canguilhem is a “philosopher of knowledge, rationality and concept” as opposed to “philosophers of experience, sense and subject” such as Satre or Merleau-Ponty. Canguilhem is an Aristotelian and this allows him to deconvolute the different contributions that convey in the medical knowledge and frame them in “a sort of structural aristotelianism (…) built of great conceptual oppositions: continuity versus discontinuity, equilibrium versus disequilibrium, vitalism versus mechanism. Any theory is inevitably partial because it can work, at any given time, with at most a single term of these basic oppositions. The great rectifications that become landmarks in scientific history are those that swing from one pole to its opposite, but the oscillation is perpetual, hence truth, while not relative, can never be absolute” [6, 15]. A second consequence is the profound reconnection with human wisdom that is lost in a technoscientific approach to medicine. In fact, despite the most complex algorithmicization, humanity remains an elusive feature to compute, reproduce or explain. It is rather an intuition and therefore superior to episteme itself according to the Platonic categorization of knowledge. The most important principle of human wisdom is the awareness of limits. Indeed, “medicine is the science of the limits of the powers that other sciences to confer upon it” [21].

We are, indeed, constrained in time and space, in abilities and power: we are mortal. This consideration is central to medicine but almost forgotten in science. Science legitimately seeks universal laws that confer control over nature and overcomes the limits that nature impose on us. Therefore, compassion is more important than – or at least complementary to—control over life events, the care of the suffering is more urgent than the elimination of sufferance – or at least as urgent as.

There is no objection to science seeking for a cure to every disease or even working toward immortality. But medicine must be practiced on the realistic ground that lifespan is limited, and disease is part of everybody’s existence. Limited lifespan is the context for medicine-techne and not the foe to fight against. In fact, medicine help us rethink time in relative terms. Oliver Sacks describes how movements and thinking may accelerate or slow-down in neurological conditions and yet appear as “just right” to the patient experiencing it [26]. The delusion of limitless time is exposed in all its absurdity by Eugene Ionesco in the play “Exit the King”, where a depressed and insecure monarch begs for more time having not realised that his over four-hundred-years long life had to come to an end [27].

Medicine-*techne* offer a framework to navigate the challenges and opportunities that science and technology can offer. While *techne* requires a primary demand from the patient, there is still space for prevention of asymptomatic/predisposing conditions. What techne has to offer is the perspective of utility, results, and benefits to the single patient in the context of the personal experience of disease. For instance, in the case of population-based screening programs, medicine-*techne* forces to reconsider the general benefits and harms in the subjective perspective of the patient; medicine is a Maieutic art. Canguilhem corroborates this view challenging the assumption that the choice to prevent – therefore eliminate – a disease, modify behaviours or health of an entire population is part of a process of “normalization of the technical means of education, health, transportation for people and goods, expresses collective demands which, taken as a whole, even in the absence of an act of awareness [prise de conscience] on the part of individuals, in a given historical society, defines its way of referring its structure, or perhaps its structures, to what it considers its own good” [4]. In light of this consideration, the Hippocratic mandate to respond primarily to patient’s subjective needs becomes a natural consequence. No norm is more acceptable than the one that each one can establish for themselves. While medicine recognizes the value of episteme in informing choices and helping patients to satisfy those needs and overcome diseases it does not enforce it. The only recognized authority is the patient, he is the origin and the scope of medicine. In Canguilhem view, there is a patient-doctor rather than a doctor-patient relationship, anticipating but keeping a distance from the Foucauldian critique to the authority of medicine^5^. In the effort to fight unnecessary medicalization, spurious data, the authoritative use of scientific data, doctors are in fact the best allied of patients. As he states: “we are not so presumptuous as to pretend to renovate medicine by incorporating a metaphysics into it. If medicine is to be renovated, it is up to physicians to do so at their risk and to their credit [4].

For Canguilhem, doctor and patient should not be seen in a cause-and-effect relationship where the action of the doctor manipulates a passive organism to cure it. Canguilhem instead recognizes the mediation of nature as a force that can be acted upon to restore health. Sometimes, he admits, the only limit is the of patients who are not keen to wait for nature to do its course [4].

## Discussion

The renewed attention to Canguilhem by an increasing number of scholars is a testament to his modernity. Last few decades have seen an exponential growth of medical knowledge and epistemic models and the challenges of translating this knowledge into clinical decision for the patients are made only more complex. Safeguarding patient autonomy and best interest requires an experiential and subjective understanding of patient needs and the ability to navigate the stratification of data, values and inference of precision medicine. Canguilhem provides a framework to negotiate Aristotelian science and art and establishes a safe hierarchy of knowledge to maximise the benefits of precision medicine.

Canguilhem’s reflections on patient-doctor relationship and self-normativity seem even more timely in the era of machine learning applied to medicine. In this perspective, Canguilhem provides a guide to safe implementation of machine learning for medical applications: his legacy is a warning against the risks of accepting the stratification of value choices and social normativity that is often embedded in the training data set and the algorithm design. A major effort of medical machine learning field is being devoted to build algorithmic models of concepts such as transparency, fairness, equity, agency and normativity. Canguilhem pointed out the role of subjectivity in medical epistemology which only recently became a major filed of investigation in artificial intelligence field known also as the alignment problem, i.e., how do we align machine learning systems to human values [7]. Yet, Canguilhem reminds us that we need to go a step further – a qualitative step—to ensure that medical knowledge and predictions generated by machine learning algorithms is aligned with each one individually. A more ambitious agenda built around Canguilhem’s medical epistemology should include the safeguard of patient’s self-normativity against a (digital) social normativity. Hard coding these principles is often challenging and comes at a cost in terms of accuracy of the algorithms. It is a necessary trade-off for a realistic representation of medical epistemology and confirms the utility of *techne* as the preferred paradigm of medical knowledge even in the era of machine learning and precision medicine.

Canguilhem criticised the whole idea of precursors in the history of science because they can only be recognised retrospectively. With the hindsight, Canguilhem has been a precursor in conceiving a medical epistemology capable to accommodate scientific discovery and objective measurement together with heuristic exploration and subjective experience. As we develop artificial intelligence for medicine we realise that all these elements must coexist to generate real-life predictive algorithms.

## Conclusions

The dialectic between art and science remains a pillar of the evolving medical practice. It is interesting to note that Canguilhem was a doctor himself, and Aristoteles was son of a reputable doctor. With their pragmatic analysis of medical epistemology, they seem to recognize the articulated process through which medicine engage with patient’s lives and ground this discipline to the anthropic principle established by the patient rather than to the positive and deterministic aspirations of science.

## Data Availability

Not applicable.

## Footnotes

1 Cangulhem’s treatise was originally published in 1966 as Le normal et le patholologique” in 1966 by Presses Universitaires de France and it was only translated in the English language in 1978 by D. Reidel Publishing Company, Dordrecht, Holland, with an introduction by Michel Foucault who was profoundly influenced by George Canguilhem.

2 “Meanwhile, Morgagni's (1682-1771) creation of a system of pathological anatomy made it possible to link the lesions of certain organs to groups of stable symptoms, such that nosographical classification found a substratum in anatomical analysis. But just as the followers of Harvey and HaIler "breathed life" into anatomy by turning it into physiology, so pathology became a natural extension of physiology. The end result of this evolutionary process is the formation of a theory of the relations between the normal and the pathological, according to which the pathological phenomena found in living organisms are nothing more than quantitative variations, greater or lesser according to corresponding physiological phenomena. Semantically, the pathological is designated as departing from the normal not so much by a- or dys- as by hyper- or hypo-. While retaining the ontological theory's soothing confidence in the possibility of technical conquest of disease, this approach is far from considering health and sickness as qualitatively opposed, or as forces joined in battle. The need to reestablish continuity in order to gain more knowledge for more effective action is such that the concept of disease would finally vanish.” [4]

3 “For Bernard, physiological and pathological processes are, qualitatively speaking, identical; that is, they differ quantitatively such that alteration from normal physiological conditions to pathological or abnormal conditions are matters of degree; all existing functions are augmented or diminished, more intense or less so than the normal condition”[28].

4 “The normal should not be opposed to the pathological, because under certain conditions and in its own way, the pathological is normal”(…) “From the biological, social, and psychological points of view, a pathological state is never a state without norms-such a thing is impossible. Wherever there is life, there are norms” [4]

5 In this respect Foucault writes: “the two dreams (i.e., nationalised medical profession and disappearance of disease) are isomorphic; the first expressing in a very positive way the strict, militant, dogmatic medicalization of society, by way of a quasi religious conversion and the establishment of a therapeutic clergy; the second expressing the same medicalization, but in a triumphant, negative way, that is to say, the volitization of disease in a corrected, organized, and ceaselessly supervised environment, in which medicine itself would finally disappear, together with its object and raison d’etre” (Birth of the Clinic).
